# Exhaled Nitric Oxide Level in Pharynx Angioedema

**DOI:** 10.3390/jcm11030637

**Published:** 2022-01-27

**Authors:** Łukasz Moos, Magdalena Zajac, Zenon Brzoza

**Affiliations:** 1 Department of Internal Diseases with Division of Allergology, Institute of Medical Sciences, University of Opole, 45-040 Opole, Poland; lukasz.moos@uni.opole.pl; 2 European Center for Diagnosis and Treatment of Urticaria (GA2LEN UCARE), 41-800 Zabrze, Poland; magdalenazajac2000@gmail.com; 3 Medical University of Wroclaw, 50-367 Wroclaw, Poland

**Keywords:** angioedema, chronic urticaria, nitric oxide

## Abstract

Airway inflammation is related to increased nitric oxide production. It can be assessed noninvasively with exhaled nitric oxide measurement. As airway inflammation was supposed to be present in chronic urticaria and angioedema patients we hypothesized increased exhaled nitric oxide in this group. Twenty-six symptomatic chronic urticaria patients with an acute episode of pharynx angioedema (17 women and 9 men, median age 35) were included in the study group. None of the patients reported a history of asthma, allergic rhinitis or cigarette smoking. The control group consisted of 29 non-smoking healthy subjects (19 women and 10 men, median age 22) without any history of atopy. Exhaled nitric oxide measurement was performed in all subjects. Exhaled nitric oxide levels in the angioedema group did not differ statistically significantly from those detected in healthy subjects (15.5 ppb and 17.0 ppb respectively). Our results indicate the lack of airway inflammation in chronic urticaria patients with pharynx angioedema.

## 1. Introduction

Nitric oxide (NO) is generated from l-arginine by different isoforms of synthases. Contrary to endothelial (eNOS or NOS-3) and neural (nNOS or NOS-1) isoforms, one form of NO synthase, i.e., inducible and calcium-dependent (iNOS or NOS-2) is not expressed in normal conditions. Constitutively expressed synthases produce NO as a result of receptor stimulation whereas iNOS is expressed in response to diverse stimuli such as inflammatory cytokines having microbidial and pro-inflammatory effects [1,2]. Constitutive forms of synthases are responsible for vasodilatation and erythema [3].

Therefore, NO may produce beneficial or adverse effects in humans. It expresses proinflammatory activity including increased vascular permeability [4]. On the other hand, when inhibiting mast cell degranulation NO shows an anti-inflammatory activity [5]. The pro- or anti-inflammatory properties of NO may vary according to different conditions [1,4].

It is well established that NO is produced by different cells such as macrophages and epithelial cells [4,6,7,8]. Recent studies show NO production in mast cells in response to different infections [9,10,11]. The exhaled NO (eNO) level is a marker of NO production in the respiratory tract [12,13,14]. Its level correlates with the intensity of airway inflammation. As it is present in the respiratory tract epithelium and cells that take part in inflammation, NO is used as a marker of type 2 airway inflammation [12,15]. The increased level of eNO -measured as fractional exhaled NO (FeNO) or alveolar concentration of NO (CaNO) was proven in patients suffering from asthma, rhinitis as well as bronchiectasis and interstitial lung disease [12,13,14,16,17]. Additionally, it was proven to be raised in patients with scleroderma and sarcoidosis, although the usefulness of FeNO and CaNO measurements in those conditions remains inconclusive [18,19]. We were the first to analyze and prove the increased nitric oxide level in patients with tonsillitis [20]. NO is hypothesized to be involved in tissue damage in autoimmune skin diseases such as psoriasis and patients with psoriatic arthritis have higher levels of FeNO than those with psoriasis only [21,22]. Acute and chronic tobacco smoking reduces FeNO levels [12].

It is still unclear whether exhaled NO levels change in a significant way during infections in asthmatic patients. Using FeNO levels seems to be unhelpful in distinguishing viral and bacterial infections. The presence of atopy (proved in children) and allergic rhinitis (both in children and adults) is associated with higher FeNO levels [12,23]. High NO production is not an effect of atopy but it results from exposure to allergens and secondary allergic inflammation [24].

Airway inflammation can be assessed noninvasively with eNO measurement. FeNO correlated significantly with bronchial hyperresponsiveness (BHR) in atopic patients with asthmatic symptoms [19]. Asero et Madonini [25] proved increased BHR in most urticaria patients, contrary to the study results of Anania and Striglia [26]. Petalas et al. [27] draw attention to the BHR in patients with cholinergic urticaria. This phenomenon is explained by the systemic release of bronchial inflammation mediators from eosinophils, basophils and mast cells [25,27,28]. Following the idea of possible BHR in urticaria patients, we hypothesized increased eNO in those patients. To date, there have been no reports on eNO level in CSU patients. Furthermore, no information is available on the relationship between upper airway angioedema and eNO. Although an inflammatory baseline or trigger is not necessary for hereditary angioedema (HAE) presentation-increased eNOS levels in serum are reported in HAE [29]. Since chronic spontaneous urticaria (CSU) and angioedema is characterized as an inflammatory disorder, we aimed to analyze eNO levels in the course of acute upper airway angioedema symptoms.

## 2. Material and Methods

We examined thirty four consecutive non-smoking chronic spontaneous urticaria patients referred to our department because of the symptoms of acute pharynx angioedema (endotracheal intubation was not required). In eNO measurement, we used Niox Mino^®^ analyser (Aerocrine AB, Solna, Sweden) with the measurement range 5 to 300 parts per billion (ppb). Its usefulness in clinical studies has already been proven [30]. The subjects were studied in the sitting position. Measurements were performed before the administration of glucocorticoids. None of the patients had been treated with steroids or antihistamine drugs in the three months preceding the analysis. Finally, 26 patients (17 women and 9 men; median age 35 years) were included in our study. On the basis of subsequent diagnostic procedures (skin prick tests, total IgE, paranasal sinuses computed tomography and ENT assessment) these patients were characterized as nonatopic and did not reveal any laboratory and clinical features of infection. In all examined patients lung function parameters were normal, none of them reported a history of asthma or allergic rhinitis. Additionally, known underlying reasons of edema (i.e., ACE inhibitors usage, C1 esterase deficiency) were excluded. The disease duration period was from 4 months to 5 years.

The control group consisted of 29 non-smoking healthy (based on medical history and physical examination) subjects (19 women and 10 men; median age 22 years). These subjects presented normal lung function parameters, did not reveal any history of atopy (confirmed with negative results of skin prick tests), and no clinical and laboratory features of airway infection.

## 3. Statistical Analysis

The statistical analysis was performed with the nonparametric U Mann–Whitney test. A *p* value of <0.05 was considered to be statistically significant.

## 4. Results

**Figure 1:**
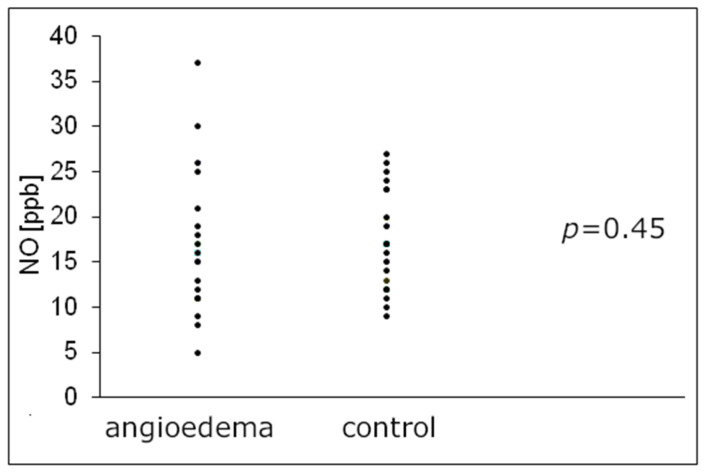
Individual exhaled nitric oxide (FeNO) level in angioedema patients in comparison to healthy subjects. FeNO—exhaled nitric oxide, NO—nitric oxide, ppb—parts per billion.

As shown in Table 1 and Figure 1, eNO levels in angioedema group did not differ statistically significantly from those detected in healthy subjects (median value 15.5 ppb and 17.0 ppb respectively; *p* = 0.45).

## 5. Discussions

Increased NO in exhaled air is a phenomenon accompanying many disorders. Besides the above-mentioned, it was proven in systemic lupus erythematosus, whereas iNOS was supposed to play an important role in Crohn’s disease and ulcerative colitis [5,31,32,33]. In non-lesional skin biopsies of patients suffering from systemic lupus erythematosus, iNOS expression in endothelial cells and keratinocytes was demonstrated [34]. NO synthase inhibition is even hypothesized to be a treatment strategy in different diseases, including those involving autoimmune mechanisms in their pathogenesis [2,5,35,36,37]. The production of NO is increased in various skin diseases (atopic dermatitis, contact dermatitis, psoriasis) [22]. In atopic dermatitis patients, the increased serum nitrate level was found, which suggests NO role in vasodilatation associated with skin erythema and edema [38].

eNOS is known to increase in patients with HAE both in the attack and in the remission phase and there are reports of significant differences in levels of other vasoactive mediators-vascular endothelial growth factors (VEGFs), angiopoietins (ANGPTs) and phospholipase A2 enzymes (PLA_2_) [29,39]. We cannot exclude that the edema of the pharynx of studied patients was due to an increased vascular permeability of eNOS as a consequence of an inflammatory state induced by proinflammatory cytokines released by mast cells.

NO level can be suppressed by corticosteroids as an effect of proinflammatory cytokine inhibition or direct influence on iNOS [40]. FeNO levels decrease in response to systemic steroid treatment of asthma exacerbation and as an effect of inhaled corticosteroid therapy [12]. Cytokines, immune complexes and complement activation products can be the factors upregulating iNOS in the epithelium, the endothelium, alveolar macrophages and smooth muscle cells [2]. In general proinflammatory cytokines, such as TNF-α, IL-1, IFN-γ and LPS induce iNOS levels [2]. On the contarary, most of FeNO in the airways is produced as a consequence of iNOS being stimulated by IL-4 and IL-13 [12,41]. Different cytokines are suspected to be involved in the pathogenesis of CSU (incl. IL-4, IL-6, IL-13, IL-17) [42,43,44,45]. Increase in IL-4 serum level was observed in both acute urticaria [46] and CSU [42]. Bae et al. [43] observed significantly higher levels of IL-13 in CSU. Among potential drugs that could be used in CSU treatment are anti-IL-4 and anti-IL-13 [47]. Decrease in different cytokines levels (including IL-4) and limiting iNOS production of NO is observed after treatment of allergic diseases with antyhistamines [48,49].

The increased eNO level in liver cirrhosis patients noticed by Matsumoto et al. [50] was hypothesized to be an effect of elevated concentrations of serum cytokines. Becherel et al. [8] proved iNOS mRNA expression in biopsies of acute urticaria lesional skin and its relation to proinflammatory cytokines [8]. They even hypothesized the role of iNOS inhibitors in the management of severe acute urticaria and some chronic forms [8]. Inducible NOS stimulation by proinflammatory cytokines that are involved in urticaria pathogenesis could result in the increased NO level in exhaled air in urticaria and angioedema patients [12,19,21,51]. Moreover, the increased eNO might be speculated as increased bronchial hyperresponsiveness related to this phenomenon [25,27,28]. Such hyperresponsiveness was supposed to be found in CSU [25].

Our results did not confirm the above hypothesis. No marked relationship between pharynx angioedema symptoms in CSU and increased FeNO levels was found. It may be speculated that the location of edema in the lower mucous membrane layers in the course of upper airway angioedema results in impaired diffusion of NO into the airways. The airway wall forms a barrier of NO diffusion [16,52]. This phenomenon is taken into account when explaining only a slight elevation in FeNO in other inflammatory disorders [12,16,52].

Pathophysiology of chronic urticaria includes wide spectrum of immune and inflammatory components, although some hormonal abnormalities and psychological components should also be taken into account [53,54,55,56].

## 6. Conclusions

The results of our study bring some more data concerning the problem of airway inflammation in chronic urticaria and angioedema patients. In our opinion, a further study concerning extended exhaled nitric oxide analysis and the NO in skin biopsies of the chronic urticaria patients should be both interesting and informative. Verifying alteration in levels of vasoactive mediators, preferably in different points in time and comparison with existing data in other conditions of similar clinical presentation could broaden the knowledge of the mechanisms underlying angioedema attack.

## Figures and Tables

**Table 1:** Exhaled nitric oxide (FeNO) level and age of angioedema patients in comparison to healthy controls.

Analysed Parameters (Unit)	Healthy Controls (N = 29)	Angioedema Patients (N = 26)	Statistical Analysis *p*
	Median Range 25–75%	Median Range 25–75%	
eNO (ppb)	17.0 9.00–27.0 13.0–20.0	15.5 5.00–37.0 11.0–19.0	0.45
age (years)	22.0 21.0–58.0 22.0–48.0	35.0 18.0–60.0 25.0–50.0	0.39

## Data Availability

The data that support the findings of this study are available from the corresponding author, (ZB), upon reasonable request.
